# Tetrahydroxy stilbene glycoside attenuates endothelial cell premature senescence induced by H_2_O_2_ through the microRNA-34a/SIRT1 pathway

**DOI:** 10.1038/s41598-022-05804-9

**Published:** 2022-02-01

**Authors:** Lixuan Zhang, Yan Guo, Shennan Shi, Yani Zhuge, Nipi Chen, Zhishan Ding, Bo Jin

**Affiliations:** 1 College of Life Science, Zhejiang Chinese Medical University, Hangzhou, 310053 Zhejiang China; 2 College of Basic Medicine, Zhejiang Chinese Medical University, Hangzhou, 310053 Zhejiang China; 3 School of Medical Technology and Information Engineering, Zhejiang Chinese Medical University, Hangzhou, 310053 Zhejiang China

**Keywords:** Cell biology, Molecular biology

## Abstract

Numerous studies have demonstrated that endothelial cell senescence plays a decisive role in the development and progression of cardiovascular diseases (CVD). Our previous results confirmed that Tetrahydroxy stilbene glycoside (TSG) can alleviate the human umbilical vein endothelial cells (HUVECs) senescence induced by H_2_O_2_ through SIRT1. It has been reported that miR-34a is a translational suppressor of SIRT1. In this study, we aimed to explore whether TSG regulates SIRT1 through miR-34a to ameliorate HUVECs senescence. H_2_O_2_ was used to induce premature senescence in HUVECs, and miR-34a mimic or inhibitor were transfected to over-express or suppress the expression level of miR-34a. Results revealed that TSG apparently decreased the miR-34a expression level in H_2_O_2_-induced premature senescence of HUVECs. When SIRT1 expression was inhibited by EX527, the attenuation of TSG on the expression level of miR-34a were abolished. When miR-34a expression was knockdown, the effect of TSG on HUVECs senescence could be enhanced. While miR-34a mimic could reverse the effect of TSG on HUVECs senescence. In conclusion, we demonstrated that TSG could attenuated endothelial cell senescence by targeting miR-34a/SIRT1 pathway.

## Introduction

With the aging of global population, the incidence of Cardiovascular disease (CVD) has increased exponentially^1^. Recent studies have shown that endothelial cells (ECs) senescence plays an important determinant role in the development and progression of CVD^2^. Therefore, exploration of effective molecules or compounds that inhibits ECs senescence may lead to enhanced prevention and treatment of CVD.

Oxidative stress plays a major role in endothelial senescence. H_2_O_2_ is a factor contributing to ECs senescence as oxidative stress. Our previous study had shown that H_2_O_2_ could trigger HUVECs senescence by down-regulating SIRT1, and Tetrahydroxy Stilbene Glycoside (TSG), a major active component of *Polygonum multiflorum* with a variety of biological effects including antioxidant and anti-inflammatory effects^3,4^, could alleviate the HUVECs senescence by up-regulating the expression of SIRT1^5^. It is well known that SIRT1 is a longevity related gene of the nicotinamide adenine dinucleotide (NAD) dependent protein family, which is believed to have the role of resistance to cell aging and vascular damage. However, the mechanism of how TSG up-regulates SIRT1 remains unclear.

MicroRNA (miRNA) is a non-coding RNA ranging between 18 and 25 nucleotides in length. Recently, the role of miRNA in regulating endothelial cell protein expression and inducing changes in vascular endothelial function has attracted widespread attention^6^. It has been reported that miR-34a increases with aging in vessels and induces senescence and the acquisition of the senescence-associated secretory phenotype (SASP) in ECs^7–9^. SIRT1 has been revealed that it could be regulated by miR-34a^10^. miR-34a inhibits SIRT1 through a miR-34a binding site with the 3’UTR of SIRT1^11,12^. Whether miR-34a is involved in the effects of TSG on ECs senescence induced by H_2_O_2_ needs further study. This study aimed to investigate that the effect of miR-34a and its target protein SIRT1 expression on the process of TSG against HUVECs senescence.

## Results

### TSG inhibited miR-34a expression in premature senescence of HUVECs induced by H_2_O_2_

**Figure 1:**
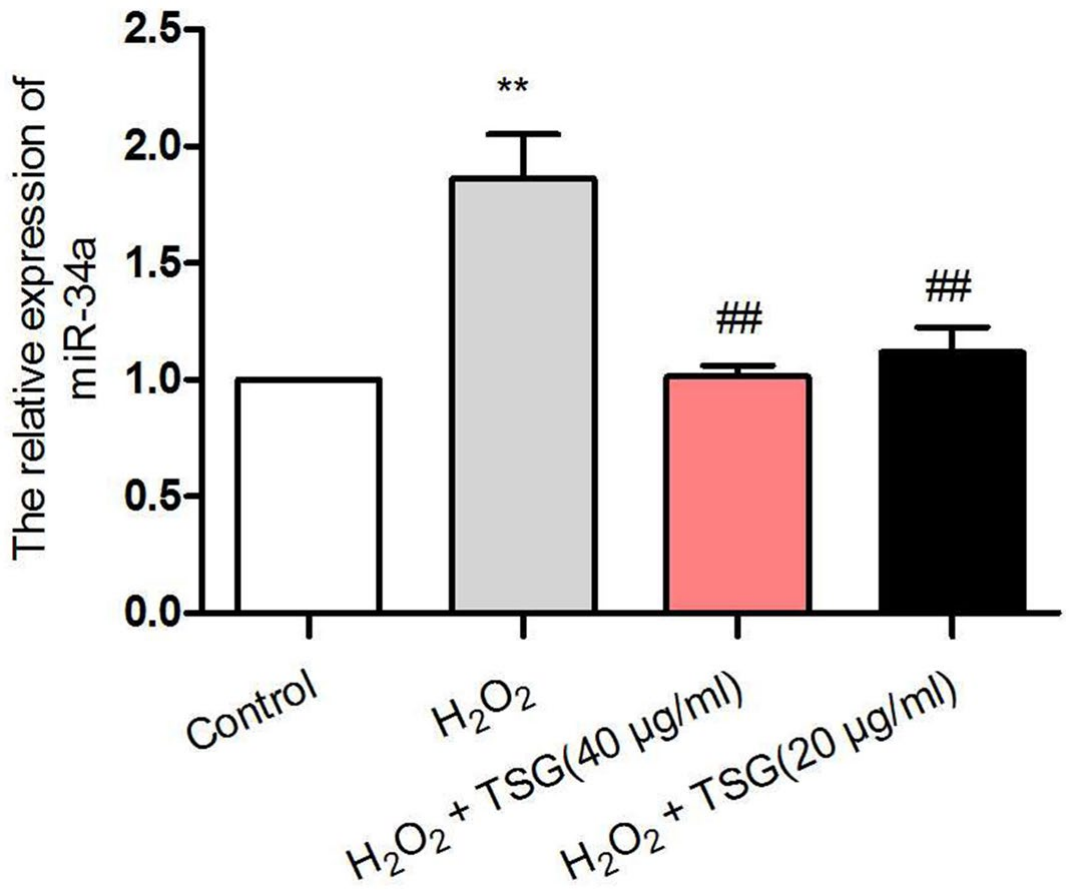
TSG inhibited miR-34a expression in H_2_O_2_-induced HUVECs. To assess the effect of TSG on miR-34a expression, after pretreatment with TSG (40 μg/ml) or TSG (20 μg/ml) for 24 h, HUVECs were exposed by H_2_O_2_ for 2 h, total RNA was extracted and the level of miR-34a was detected by qRT-PCR. Results are expressed as the means ± SD. ^**^p < 0.01 vs. control, ^*##*^*p* < 0.01 vs. H_2_O_2_ group.

To clarify the effect of TSG on miR-34a, TSG (40 μg/ml, 20 μg/ml) was administered to the H_2_O_2_-induced HUVECs. qRT-PCR analyses (Fig. 1) indicated that miR-34a was elevated upon stimulation with H_2_O_2_. Compared with the H_2_O_2_ group, it led to a significantly decreased expression of miR-34a with TSG pretreatment. And the 40 μg/ml of TSG had been used for subsequent experiments.

**Figure 2:**
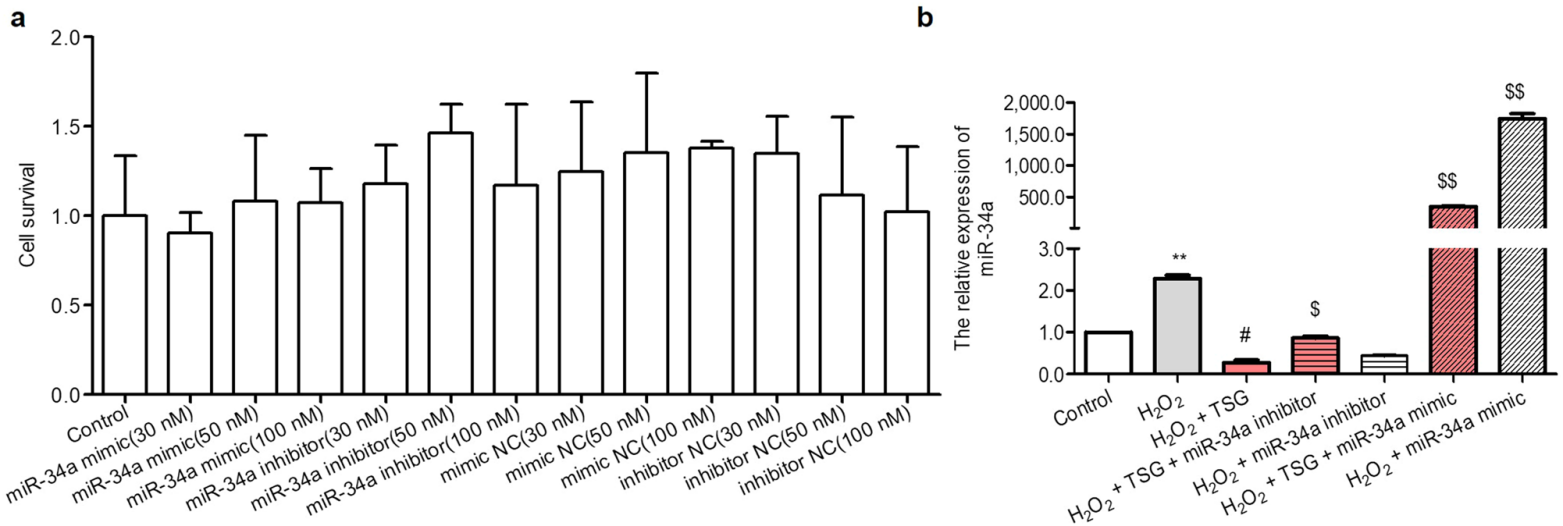
The effect of TSG on the expression level of miRNA-34a in H_2_O_2_-induced HUVECs when over-expression or suppression of miR-34a. (**a**) Effects of different concentrations of the miR-34a mimic or the miR-34a inhibitor and their negative control (mimic NC, inhibitor NC) on the percentage of cell viability in normal HUVECs. Cell viability was detected by the MTS assay. (**b**) HUVECs were transfected with miR-34a mimic or miR-34a inhibitor for 24 h, then treated with TSG 24 h, H_2_O_2_ was added and incubated for 2 h. Total RNA was extracted and the level of miR-34a was detected by qRT-PCR. Results are expressed as the means ± SD. ^**^p < 0.01 vs. control, ^*#*^*p* < 0.05 vs. H_2_O_2_ group, ^*$*^*p* < 0.05 and ^*$$*^*p* < 0.01 vs. H_2_O_2_ + TSG group.

To further confirm the interaction of TSG and miR-34a in HUVECs senescence, miR-34a mimic, miR-34a inhibitor and their negative control (mimic NC, inhibitor NC) were transfected into H_2_O_2_-induced HUVECs. First, several concentrations of miR-34a mimic/mimic NC (30, 50, 100 nM) and miR-34a inhibitor/inhibitor NC (30, 50, 100 nM) were transfected into HUVECs to verify the optimal transfection. In addition, the results of cell viability by MTS illustrated that miR-34a mimic/mimic NC (30, 50, 100 nM) and miR-34a inhibitor/inhibitor NC (30, 50, 100 nM) had no significant cytotoxicity on HUVECs (Fig. 2a). Subsequently, the expression of miR-34a could be efficiently over-expressed or inhibited by transfection with miR-34a mimic (50 nM) or miR-34a inhibitor (100 nM), respectively (Fig. 2b). Compared with TSG pretreatment only, miR-34a mimic significantly blocked the inhibitory effect of TSG on miR-34a. Meanwhile, in the presence of TSG in H_2_O_2_-induced HUVECs, the inhibition of miR-34a could strengthen the inhibitory effect of TSG. Hence, miR-34a could be significantly down-regulated by TSG in H_2_O_2_-induced HUVECs.

### TSG inhibited miR-34a causing the activation of SIRT1

**Figure 3:**
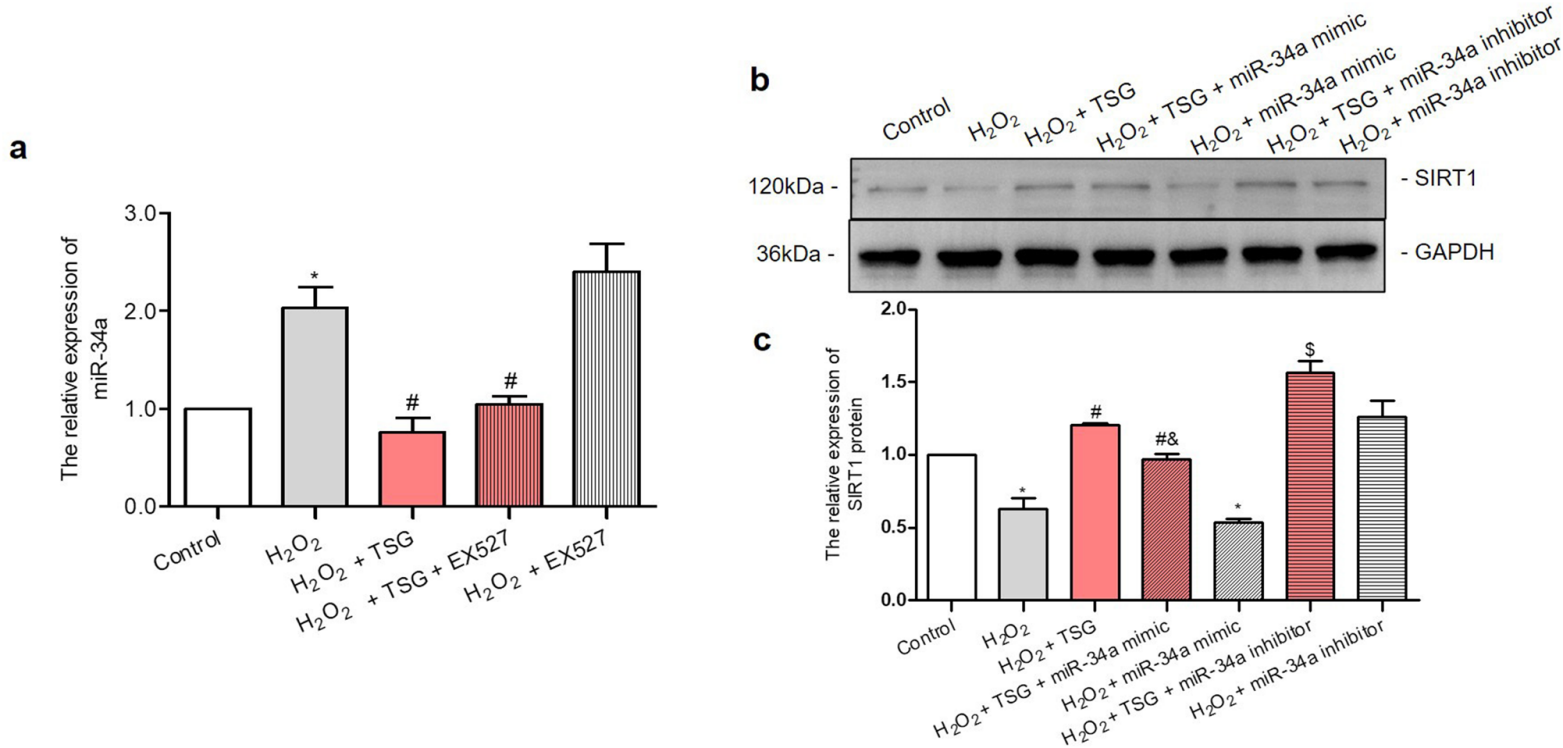
TSG down-regulated miR-34a resulting the activation of SIRT1. To study the mediating role of miR-34a in the regulation of SIRT1 by TSG, EX527 was used to inhibit SIRT1, and miR-34a mimic or inhibitor were used to interfere with miR-34a. (**a**) The effect of TSG pretreatment on miR-34a expression after SIRT1 inhibition by EX527 in H_2_O_2_**-**induced HUVECs. Except for the control group, all cell groups were treated with H_2_O_2_. Before H_2_O_2_ induction, HUVECs were pretreated with TSG, EX527 or TSG + EX527 respectively for 24 h, the expression level of miR-34a was determined by qRT-PCR. (**b, c**) The effect of TSG pretreatment on SIRT1 expression after miR-34a over-expression or suppression. HUVECs were transfected with miR-34a mimic or miR-34a inhibitor for 24 h, then treated with TSG for 24 h, and finally induced by H_2_O_2_, the expression level of SIRT1 was determined by Western Blot. GAPDH was used as an endogenous control for SIRT1 expression. All groups of cells were treated with H_2_O_2_ before administrations expect control group. Uncropped gel images are provided in the supplementary information. ^*^p < *0.05* vs. control, ^*#*^*p* < *0.05* vs. H_2_O_2_ group, ^*$*^*p* < *0.05* vs. H_2_O_2_ + TSG group, ^*&*^*p* < *0.05* vs. H_2_O_2_ + miR-34a mimic group.

Studies have shown that miR-34a family was an upstream regulator of SIRT1. To determine whether the regulating effects of TSG on SIRT1 in H_2_O_2_-induced HUVECs were mediated by miR-34a, the HUVECs were treated with H_2_O_2_, in the presence of TSG, TSG and SIRT1 inhibitor EX527 or EX527. As shown in Fig. 3a, TSG group significantly inhibited miR-34a expression enhanced by the SIRT1 inhibitor EX527. Next, the following study explored the effect of miR-34a mimic or inhibitor on SIRT1 within TSG treatment in H_2_O_2_-induced HUVECs. As shown in Fig. 3b, c, compared with the H_2_O_2_ group, SIRT1 protein was less expressed in the miR-34a mimic group, and TSG elevated the expression of SIRT1 reduced by miR-34a mimic. While compared with the H_2_O_2_ + TSG group, SIRT1 expression was up-regulated after the adding miR-34a inhibitor. This demonstrated that miR-34a inhibitor increased SIRT1 protein level, and enhanced the promotion effect of TSG on SIRT1. These results indicated that in H_2_O_2_-induced premature HUVECs, TSG increased SIRT1 by inhibiting miR-34a expression.

### TSG suppressed the apoptosis in H_2_O_2_-induced HUVECs mediated by miR-34a

**Figure 4:**
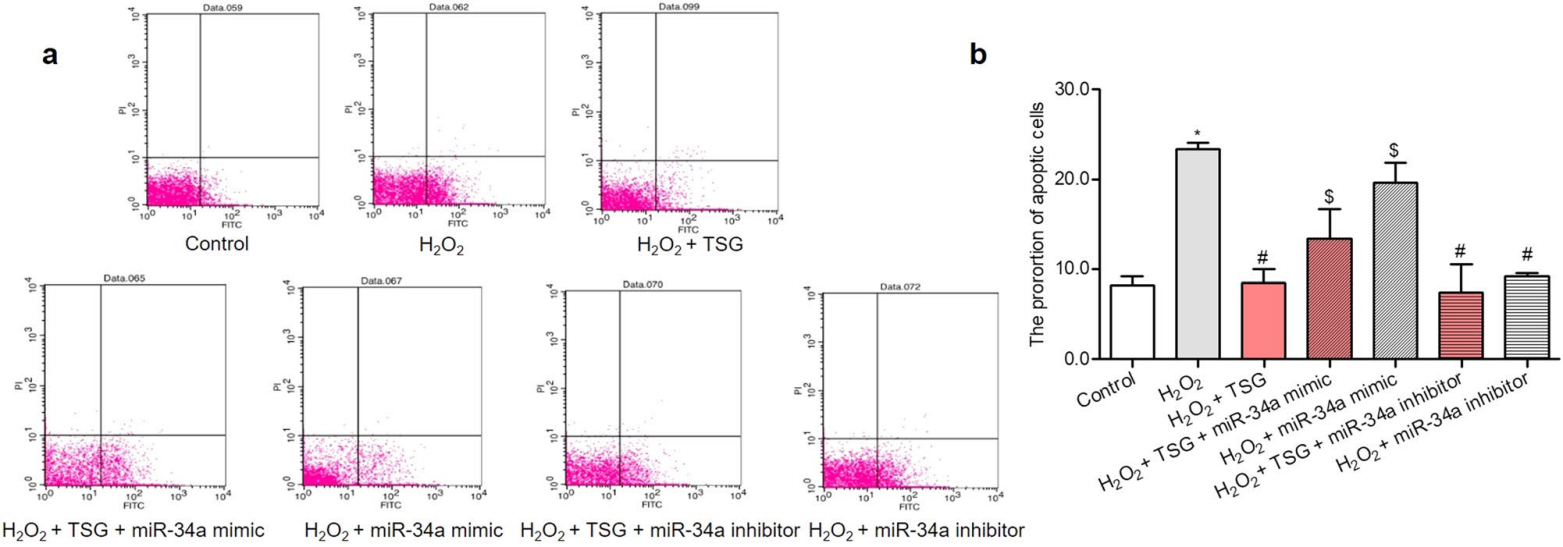
The effect of miR-34a over-expression or suppression on apoptosis rate of TSG on HUVECs induced by H_2_O_2_. HUVECs were transfected with miR-34a mimic or miR-34a inhibitor for 24 h, then treated with TSG for 24 h, and each group was finally induced by H_2_O_2_, except control group. The cells samples were analyzed by Fluorescein-conjugated Annexin V (Annexin V-FITC) vs propidium iodide (PI) staining, apoptotic cells were determined as Annexin V-FITC positive and PI negative. The cells located in the lower right quadrant were early apoptosis cells. (**a**) Representative flow plot of early apoptosis distribution. (**b**) The data were normalized to control. ^*^p < *0.05* vs. control, ^*#*^*p* < *0.05* vs. H_2_O_2_ group, ^*$*^*p* < *0.05* vs. H_2_O_2_ + TSG group.

In order to determine the effects of miR-34a on TSG inhibiting the HUVECs apoptosis, we analyzed the proportion of apoptosis cells when miR-34a was over-expressed or down-expressed. As shown in Fig. 4a, b, compared with the H_2_O_2_ group, the TSG and miR-34a inhibitor alone could significantly inhibit cell apoptosis, and the combination of them had an enhanced effect. Whereas, compared with H_2_O_2_ + TSG group, over-expression of miR-34a by miR-34a mimic increased the number of apoptosis cells, and could significantly reverse the down-regulation of TSG on cell apoptosis. These data indicated that TSG alleviated cell apoptosis through miR-34a.

### miR-34a mediated the effect of TSG on H_2_O_2_-induced HUVECs’ cell cycle

**Figure 5:**
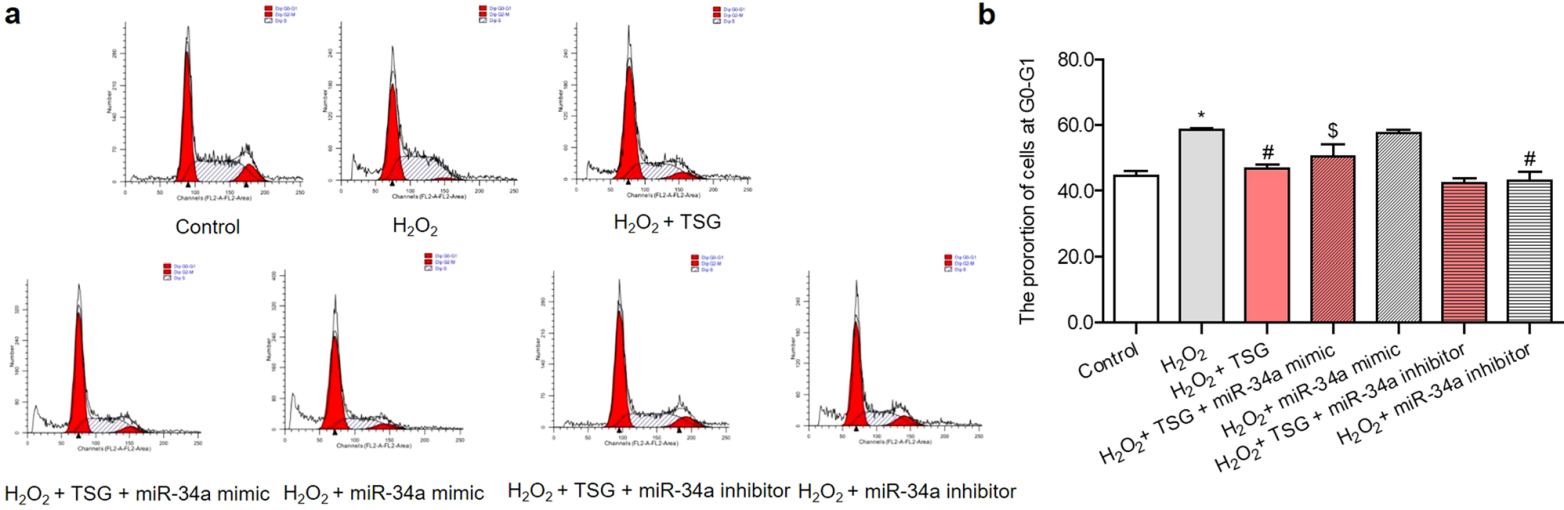
The effect miR-34a over-expression or suppression on cell cycles of TSG on HUVECs induced by H_2_O_2_. HUVECs were transfected with miR-34a mimic or miR-34a inhibitor for 24 h, then treated with TSG for 24 h, and each group was finally induced by H_2_O_2_, except control group. The cells samples were stained by PI staining, and flow cytometry analysis was used to detected the quantification of cell cycle proportion. (**a, b**) Representative flow plot of cell cycle distribution. ^*^p < *0.05* vs. control, ^*#*^*p* < *0.05* vs. H_2_O_2_ group, ^*$*^*p* < *0.05* vs. H_2_O_2_ + TSG group.

To further evaluate the protective effect of miR-34a on TSG alleviating HUVECs senescence, miR-34a mimic or inhibitor were applied to TSG-treated H_2_O_2_-induced HUVECs in order to verify whether miR-34a mediated the effect of TSG on the cell cycle. The results showed that TSG could cut down the proportion of G0-G1 stage cells induced by H_2_O_2_. Compared with the H_2_O_2_ group, miR-34a inhibitor had an inhibitory effect on stopping the cell cycle in the G0-G1 phase, similar as TSG (Fig. 5a, b). Meanwhile, compared with H_2_O_2_ + TSG group, miR-34a mimic could significantly reverse the percentage of G0-G1 cells decreased by TSG. Therefore, the results indicated that TSG could reduce the percentage of cells in the G0-G1 phase through inhibiting the expression of miR-34a.

### Protective effects of TSG against H_2_O_2_-induced HUVECs aging regulating miR-34a/SIRT1 signaling pathway

**Figure 6:**
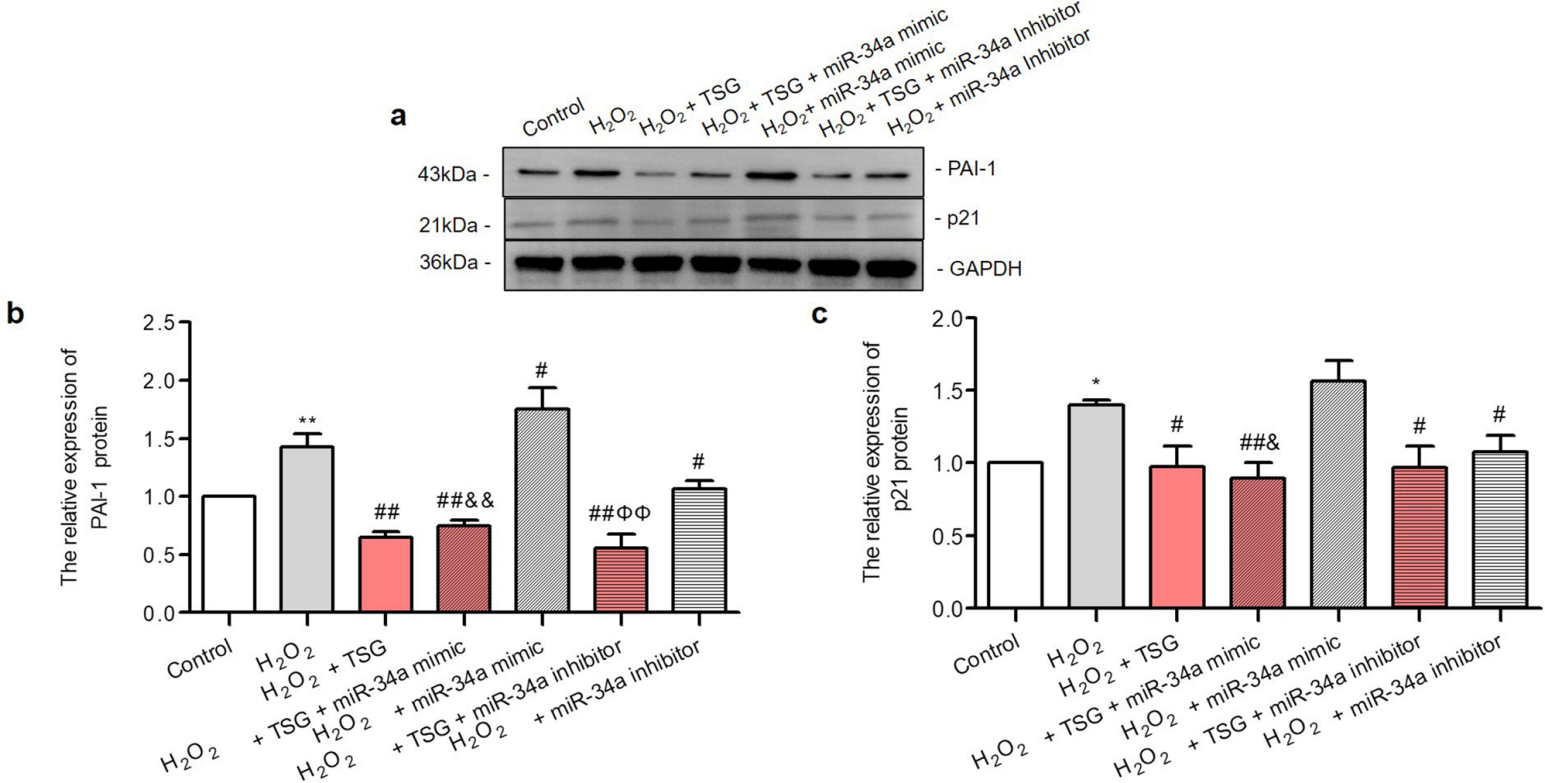
The effect of miR-34a over-expression or suppression on cell senescence related protein expression level of TSG on HUVECs induced by H_2_O_2_. HUVECs were transfected with miR-34a mimic or miR-34a inhibitor for 24 h, then treated with TSG for 24 h, and each group was finally induced by H_2_O_2_, except control group. (**a**) Representative images of the semi-quantification of p21, PAI-1 and GAPDH determined by Western Blot. GAPDH was used as an endogenous control. (**b**) The expression level of PAI-1 in each group. (**c**) The relative expression of p21 protein in each group. All groups of cells were treated with H_2_O_2_ expect control group. Uncropped gel images are provided in the supplementary information. ^*^p < *0.05* and ^**^p < *0.01* vs. control, ^*#*^*p* < *0.05* and ^*##*^*p* < *0.01* vs. H_2_O_2_ group, ^*$*^*p* < *0.05* vs. H_2_O_2_ + TSG group, ^*&*^*p* < *0.05* and ^*&&*^*p* < *0.01* vs. H_2_O_2_ + miR-34a mimic group, ^*ϕϕ*^*p* < *0.01* vs. H_2_O_2_ + miR-34a inhibitor group.

Our previous result suggested that TSG attenuated H_2_O_2_-induced HUVECs cell senescence by regulating SIRT1. To further study the effect of miR-34a on endothelial dysfunction and senescence, we analyzed the expression of senescence-associated proteins when miR-34a was over-expressed or suppressed, such as PAI-1 and p21 proteins. Compared with the H_2_O_2_ group (Fig. 6a–c), we found that the PAI-1 and p21 protein levels were significantly decreased by TSG, and miR-34a inhibitor had the similar effect. In contrast, miR-34a mimic significantly increased the PAI-1 expression level. Compared with miR-34a mimic group, TSG could cause a significant decrease in PAI-1 and p21 expression increased by miR-34a mimic. Compared with miR-34a inhibitor group, TSG significantly promoted the inhibition effect of miR-34a inhibitor on PAI-1. TSG could further promote the miR-34a inhibitor down-regulation of p21, but the difference was not significant. Thus, these results showed that TSG attenuated cellular senescence through miR-34a.

## Discussion

Cell senescence is a process of cellular physiological function decline caused by oxidative stress. Aging of endothelial cells will disrupt the cell balance of the body, leading to endothelial dysfunction, which is a major risk factor for accelerating the development of cardiovascular diseases^13–15^. Therefore, anti-endothelial cell senescence is of great significance to the prevention and treatment of cardiovascular diseases. TSG has been recognized as a potential therapeutic agent in the treatment of cardiovascular-related diseases^16^. We recently published that TSG exerted inhibition effect on H_2_O_2_-induced senescence in HUVECs^5^. In this present study, we demonstrated the protective mechanism that TSG inhibited cell senescence via the miR-34a/SIRT1 signal axis.

miRNA is a class of small non-coding RNA that could regulate gene expression levels during multiple cellular processes^17^. miRNA, primarily acting as a negative regulator of mRNA translation, is involved in the regulation of cell cycle regulation, stress response, differentiation, aging, apoptosis by binding to the complementary sequences in the 3’untranslated region (3’UTR)^18^. Thus, aberrant expression of miRNA affects multiple biological processes, including cell differentiation, proliferation, and apoptosis^19^. More importantly, certain miRNA have been shown to be important regulators of aging-related gene expression and play an important role in cell senescence^20^. Based on previous studies, miR-34a is up-regulated in many aging-related diseases^21^. Li et al.^22^ and Cui et al.^23^ have proved that miR-34a plays a key role in the process of cell senescence as a pro-senescence factor. Consequently, we detected the activation status of miR-34a genes during the induction of cell senescence in this experiment. Our results showed that miR-34a was highly expressed in H_2_O_2_-induced cell senescence model, indicating miR-34a elevated is associated with pathological events of senescence in vascular endothelial cells, which was consistent with the above research conclusions. Notably, this effect was regulated by TSG treatment. This prompted us to further explore the role of miR-34a in TSG in the treatment of cell senescence.

To test our speculation of miR-34a crucially contributing to the anti-aging effects of TSG. We modulated miR-34a expression in H_2_O_2_-induced premature senescence HUVECs. As expected, we found that miR-34a over-expression in HUVECs markedly induced senescence related alternations, such as increased apoptosis, retarded cell cycle progression with the majority of cells arrested in G1, whereas TSG treatment improved it, similar as miR-34a inhibitor. PAI-1 is a serine protease inhibitor, which is a mediator and marker of cell aging^24^. Studies have shown that PAI-1 is highly expressed in senescent cells, and there is a direct correlation between increased PAI-1 level and senescence related galactosidase-positive cells. p21 is a main cell cycle regulator which can bind cyclin/CDK complexes and regulate cell cycle progression negatively^25^. In our study, compared with that in H_2_O_2_-induced cell senescence group, the TSG group and the miR-34a inhibitor group had similar effects, which could down-regulate the contents of senescence related factors PAI-1 and p21. With all the above results, we suggested that the protective effect of TSG on cellular senescence in H_2_O_2_-induced premature HUVECs could be compensated through the down-regulation of miR-34a.

Studies have confirmed that the activation of miR-34a/SIRT1/p53 signaling was involved in endothelial cell dysfunction and apoptosis^26^. Our previous studies had confirmed that TSG could prevent HUVECs from senescence by activating SIRT1 and decreasing p53 expression^5^. It has been demonstrated that miR-34a negatively regulates SIRT1 expression, which is also reflected by our study, where miR-34a expression was significantly enhanced under the intervention of the SIRT1 inhibitor EX527. The function of miR-34a/SIRT1 pathway in H_2_O_2_-induced cell damage has been demonstrated by Guo et al.^27^. Here, our results indicated that TSG up-regulated SIRT1 while suppressed miR-34a expression in HUVECs, and these disturbances were inhibited by EX527 and miR-34a mimic. Conversely, miR-34a inhibitor could enhance the up-regulation of SIRT1 by TSG. With the combination of TSG and miR-34a inhibitor, it showed higher SIRT1 expression in HUVECs, compared to cells treated with TSG alone. Collectively, these results strongly supported that activated miR-34a/SIRT1 signaling was required for TSG to intervene in HUVECs premature senescence induced by H_2_O_2_.

In conclusion, our current study has revealed that TSG inhibited HUVECs premature senescence induced by H_2_O_2_ through targeting miR-34a/SIRT1. However, our data were mainly obtained from the in vitro model of HUVECs, deeper studies on delineating the anti-aging function of TSG and the control of miR-34a/SIRT1 network on endothelial cell senescence through in vivo methods needs to be elucidated later, because there are other aging-related regulatory mechanisms that may also be involved. Additionally, the interaction between TSG and miR-34a is also worth dedicated studies.

## Conclusions

Collectively, our data suggested that miR-34a/SIRT1 pathway was involved in the TSG inhibiting aging process of HUVECs. TSG may be used as a promising therapy drug to reduce vascular senescence and aging.

## Methods

### Materials

The HUVECs were purchased from the Institute of Cell Biology, Chinese Academy of Sciences. The Tetrahydroxy Stilbene Glycoside (TSG, CAS:82373-94-2, purity: ≥ 98%) and the EX527 (CAS:49843-98-3, purity: ≥ 98%) were purchased from Sigma-Aldrich Co. LLC, American. The micrON™ hsa-miR-34a-5p mimic/micrON™ mimic Negative Control and micrOFF™ hsa-miR-34a-5p inhibitor/micrOFF™ inhibitor Negative Control were purchased from Riobio, Chinese. And TSG was dissolved in sterile water.

### Cell culture and treatment

HUVECs were cultured in RPMI 1640 medium (Gino, Hangzhou, China) containing 10% heat-inactivated fetal bovine serum (FBS, Sigma-Aldrich, American) and 1% penicillin–streptomycin at 37 °C in 5% CO_2_.

The cells were randomly divided into groups. Control group: HUVECs were cultured with normal medium. H_2_O_2_ group: HUVECs were exposed to H_2_O_2_ (200 μM) for 2 h and recovered for 24 h. H_2_O_2_ + TSG group: HUVECs were pretreatment with TSG (40 μg/ml) for 24 h before H_2_O_2_ inducing. H_2_O_2_ + TSG + miR-34a mimic or miR-34a inhibitor group: HUVECs were transfected with miR-34a mimic (50 nM) or miR-34a inhibitor (100 nM) for 24 h, then treated the HUVECs as the TSG group. H_2_O_2_ + miR-34a mimic, miR-34a inhibitor or EX527 group: HUVECs were transfected with miR-34a mimic (50 nM), miR-34a inhibitor (100 nM) or EX527 for 24 h, prior to the H_2_O_2_ inducing. H_2_O_2_ + TSG + EX527 group: HUVECs were pretreated with EX527 (40 μM) for 24 h, then treated the HUVECs as the TSG group.

### MTS assay

HUVECs were cultured in 96-well plates at a density of 3,000 per well for 24 h. And then they were treated with different treatment factors according to the grouping requirements. Cells were incubated with 20 μl MTS (Beyotime, China) for 2 h at 37 ℃ to measure their growth. The absorbance of each well was quantified at 490 nm. There were 6 multiple holes in a single group.

### Isolation of RNA and real-time quantitative RT-PCR analysis (qRT-PCR)

For analysis of miRNA and Total RNA were extracted by TRIzol (Invitrogen). The first strand cDNA was synthesized using PrimeScript RT Master Mix (TaKaRa) at 37 ℃, 60 min, 85 ℃, 5 min, stored at 4 ℃. qPCR was performed with SYBR Premix Ex TaqTM II (TaKaRa) according to the manufacturer’s instructions. Gene relative quantitative was analyzed by 2^-ΔΔCt^ method and all analyses were performed in triplicate. The primer sequences used in this study were as follows: miR-34a: TGGCAGTGTCTTAGCTGGTTGT, U6: ATTGGAACGATACAGAGAAGATT.

### Western blot analysis

RIPA lysis buffer was added to the collected cells and the concentration was measured with a bicinchoninic acid (BCA) kit (Beyotime, China). Protein samples were separated by SDS-PAGE using a 10% polyacrylamide gel. Then membranes were exposed to anti-SIRT1 (1:1,000, CST), anti-p21 (1:2,000, Proteintech), anti-PAI-1 (1:1,000, CST), and anti-GAPDH (1:5,000, Proteintech) overnight at 4 ℃. The membranes were washed (three times, 10 min each) in Tris-buffered saline (TBS) containing 0.1% Tween-20 (TBST) and then incubated with the corresponding secondary antibody. Based on the protein band needed, the PVDF membrane was cut prior to antibody hybridization according to the protein size. Band intensity was quantified by Image J software.

### Cell cycle analysis

The cell cycle status was quantitatively analyzed by flow cytometry-based on propidium iodide (PI) staining. Cells were collected and fixed overnight with 70% alcohol at 4 ℃. The cells were centrifuged to remove the alcohol and washed twice with PBS (4 ℃). Next, the PI stain was added, and the cells were protected from light for 30 min at 4 ℃. The samples were analyzed by BD FACSCalibur (BD Bioscieces, USA). Analysis of cell cycle distribution was performed with BD FACSCalibur software.

### Annexin V-FITC/PI dual staining

Cell apoptosis analysis was performed using the Annexin V-FITC Apoptosis Detection Kit (Beyotime). Briefly, after experimental treatments, the cells were washed twice with PBS (4 ℃) and resuspended in 100 ml binding buffer, followed by incubation with 5 μl Annexin V-FITC and 10 μl PI at room temperature for 15 min. A total of 10,000 cells were collected and analyzed by BD FACSCalibur (BD Biosciences, USA).

### MiRNA transfection

Cells in the exponential phase of growth were plated in six-well plates at 2 × 10^5^ cells/plate and cultured for 24 h. Then, the cells were transfected with the miR-34a mimic (50 nM) and the miR-34a inhibitor (100 nM) using Lipofectamine RNAimax transfection reagent (Invitrogen) according to the manufacturer's protocols.

### Statistical analysis

The data were presented as mean ± SD using GraphPad Prism software. For multiple comparisons, one-way ANOVA followed by Tukey post hoc test was performed. *p* < *0.05* was considered significant.

## Supplementary Information

Supplementary Information.

## Data Availability

The datasets generated for this study are available on request to the corresponding author.
