# Supplementary material for: Tetrahydroxy stilbene glycoside attenuates endothelial cell premature senescence induced by H2O2 through the microRNA-34a/SIRT1 pathway
Source: Sci Rep. 2022 Feb 1;12:1708. doi: 10.1038/s41598-022-05804-9 (PMC8807705; doi:10.1038/s41598-022-05804-9)
Supplement: Supplementary file 1 — Supplementary Information. [file 41598_2022_5804_MOESM1_ESM.pdf]

MicroRNA-34a/SIRT1: A pathway for Tetrahydroxy stilbene glycoside to attenuate endothelial cell premature senescence induced by H_2O_2

Lixuan Zhang [1#], Yan Guo[2#], Shennan Shi[1], Yani Zhuge[1], Nipi Chen[1], Zhishan Ding[3*], Bo Jin[1*]

[1] College of Life Science, Zhejiang Chinese Medical University, Hangzhou, 310053, Zhejiang, China

[2] College of Basic Medicine, Zhejiang Chinese Medical University, Hangzhou, 310053, Zhejiang, China

[3] School of Medical Technology and Information Engineering, Zhejiang Chinese Medical University, Hangzhou, 310053, Zhejiang, China

Lixuan Zhang: zlx979185105@163.com
Yan Guo: joygy1401@163.com
Shennan Shi: shishennan0108@163.com
Yani Zhuge: zhugeyani010413@163.com
Nipi Chen: nebe323@zcmu.edu.cn
Zhishan Ding: dzszjtcm@163.com
Bo Jin: jinbo@zcmu.edu.cn
*Correspondence: Bo Jin: jinbo@zcmu.edu.cn, or Zhishan Ding: dzszjtcm@163.com
#Contributed equally to this work

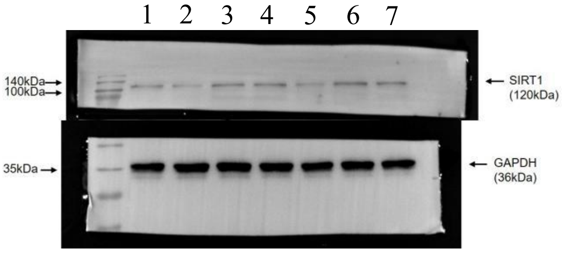

1.Control

2.H_2O_2

3.H_2O_2+TSG

4.H_2O_2+TSG+miR-34a mimic

5.H_2O_2+miR-34a mimic

6.H_2O_2+TSG+miR-34a inhibitor

7.H_2O_2+miR-34a inhibitor

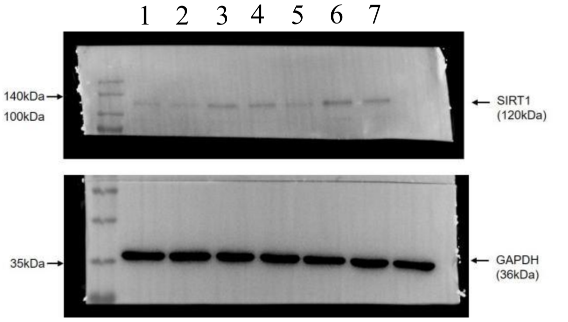

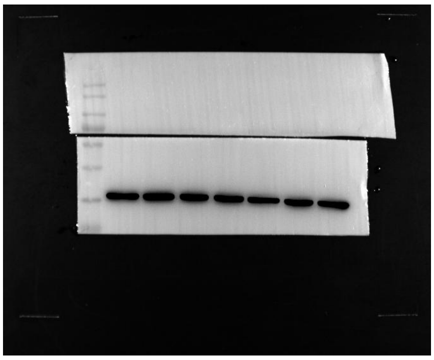

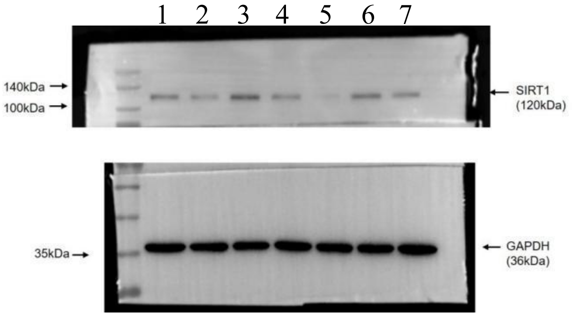

Represented gel blot images of Figure 3b

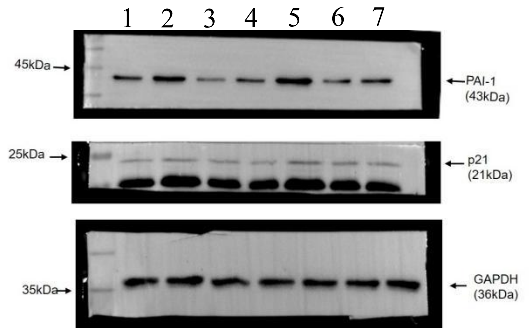

1.Control
2.H_2O_2
3.H_2O_2+TSG
4.H_2O_2+TSG+miR-34a mimic
5.H_2O_2+miR-34a mimic
6.H_2O_2+TSG+miR-34a inhibitor
7.H_2O_2+miR-34a inhibitor

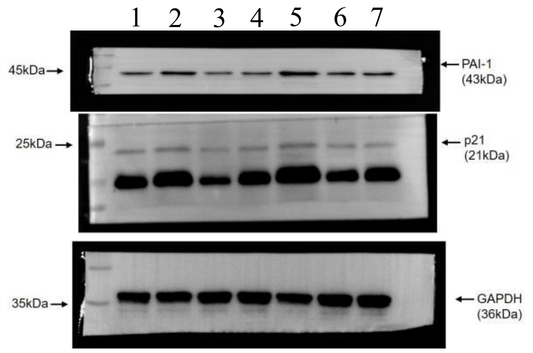

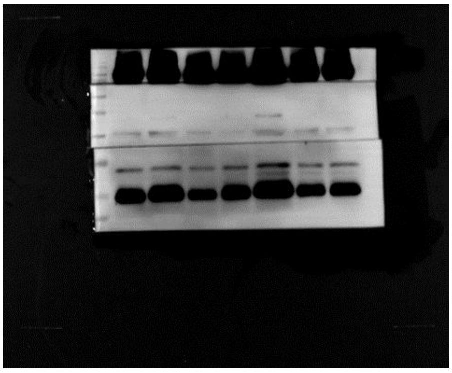

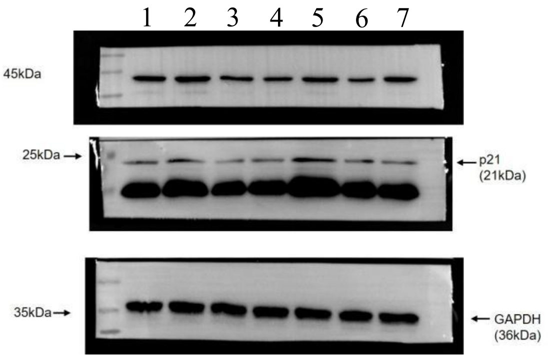

Represented gel blot images of Figure 6a
